# Limited Emergence of *Salmonella enterica* Serovar Infantis Variants with Reduced Phage Susceptibility in PhagoVet-Treated Broilers

**DOI:** 10.3390/ani14162352

**Published:** 2024-08-14

**Authors:** Sandra Sevilla-Navarro, Jennifer Otero, Júlia López-Pérez, Jan Torres-Boncompte, Tiago Prucha, Maarten De Gussem, Daniela Silva, Julia Burgan, Pablo Catalá-Gregori, Pilar Cortés, Montserrat Llagostera

**Affiliations:** 1 Centro de Calidad Avícola y Alimentación Animal de la Comunidad Valenciana (CECAV), 12539 Alquerias NP, Castellón, Spain; s.sevilla@cecav.org (S.S.-N.); j.torres@cecav.es (J.T.-B.); p.catala@cecav.org (P.C.-G.); 2 Molecular Microbiology Group, Departament de Genètica i de Microbiologia, Universitat Autònoma de Barcelona, 08193 Cerdanyola del Vallès, Barcelona, Spain; jennifer.otero@uab.cat (J.O.); julia.lopez@uab.cat (J.L.-P.); montserrat.llagostera@uab.cat (M.L.); 3 VETWORKS BVBA, Knokstraat 38, 9880 Poeke, Belgium; tiago.prucha@vetworks.eu (T.P.); maarten.degussem@vetworks.eu (M.D.G.); 4 ALS Life Sciences Portugal, Zona Industrial de Tondela ZIM II lote 6, 3460-070 Tondela, Portugal; daniela.silva@alsglobal.com (D.S.); juliaburgan@gmail.com (J.B.)

**Keywords:** *Salmonella* Infantis, broilers, bacteriophage, PhagoVet, reduced phage susceptibility

## Abstract

**Simple Summary:**

One of the main concerns associated with phage applications is the potential emergence of bacterial variants exhibiting reduced susceptibility to bacteriophages, which may jeopardize the success of such applications, as occurs with antibiotics. In this work, we studied the emergence of reduced-phage-susceptibility variants in broiler trials challenged with *Salmonella enterica* serovar Infantis and treated with the PhagoVet product. We characterized the bacteriophages composing the PhagoVet product at both a microscopic and genomic level, which displayed a broad host range of infection against 271 strains representing 18 *Salmonella* serovars. Our results indicate that the emergence of reduced-phage-susceptibility variants is unlikely to compromise the efficacy of oral PhagoVet against *S*. Infantis.

**Abstract:**

*Salmonella enterica* serovar Infantis (*S*. Infantis) poses a growing issue in the poultry sector, with phage-based products emerging as a safe and effective control measure. This study investigated the emergence of reduced-phage-susceptibility variants (RPSV) of *S*. Infantis in PhagoVet-treated broilers, given that RPSV could undermine phage treatment efficacy. The bacteriophages in the PhagoVet product were characterized using transmission electron microscopy (TEM), genome sequencing, and infection profiling. Furthermore, two broiler trials were conducted: a challenge group (T1) and a challenge-and-treated group (T2). The *S*. Infantis infective dose was set at 10^4^ and 10^6^ colony-forming units (CFUs) per animal, with PhagoVet administration at 10^6^ and 10^8^ plaque-forming units (PFUs) per animal, in Trials 1 and 2, respectively. The results revealed that the four PhagoVet bacteriophages belonged to different genera. PhagoVet evidenced broad-spectrum efficacy against 271 strains representing 18 *Salmonella* serovars. In Trial 1, PhagoVet reduced bacterial counts in feces to nearly undetectable levels by day 42, with no RPSV detected. However, in Trial 2, three and five RPSVs were detected in feces and ceca, respectively. Consequently, PhagoVet demonstrated efficacy against *S*. Infantis in broilers, and the potential impact of RPSV is deemed unlikely to compromise its efficacy.

## 1. Introduction

*S*. Infantis currently ranks among the top 10 serovars associated with human infections, standing as the fourth leading cause of salmonellosis cases in the EU [1]. Recent data from the European Food Safety Authority (EFSA) indicated that 95.6% of the *Salmonella* isolates in broiler flocks belong to the Infantis serovar, demonstrating a close association with poultry production [2]. In recent years, the increasing incidence of *S*. Infantis infections in both humans and animals has been further complicated by the dissemination of multidrug-resistant (MDR) clones across several countries. In fact, these MDR strains have been linked to prolonged illness, extended hospitalizations, and increased mortality rates, thereby posing considerable public health implications [3]. Alarming levels of AMR (45.3%) have been reported in *S*. Infantis strains isolated from broilers, particularly against sulfonamides, tetracyclines, ciprofloxacin, and cefotaxime, antibiotics classified by the World Health Organization (WHO) as of “critical importance & highest priority” for human medicine due to limited alternative treatment options [1,4,5,6,7].

*S*. Infantis exhibits distinct genetic characteristics, most of them encoding the pESI-like mega-plasmids, that enhance its epidemiological fitness, particularly in terms of easy acquisition and transmission of antimicrobial resistance (AMR), resistance to heavy metals, possession of mobile virulence genes, and proficiency in biofilm formation [3,6,7,8]. These attributes have established *S*. Infantis as a widely distributed serovar with persistent infections in animal production, particularly in the poultry sector.

Since 2007, the poultry sector has implemented stringent cleaning and disinfection protocols, biosecurity measures, and prophylactic interventions. While these measures have been demonstrated to be effective against *Salmonella* Enteritidis and Typhimurium, challenges have been encountered in the case of *S*. Infantis due to the unavailability of authorized live vaccines and the bacterium’s high tolerance and adaptation to current chemical solutions. This has resulted in the failure of the cleaning and disinfection processes [9]. Therefore, finding effective tools for the prevention and control of *S*. Infantis, such as the use of bacteriophages, is imperative. 

Bacteriophages, or phages, stand out as one of the safest options for the prevention, treatment, and eradication of bacterial pathogens including MDR ones. Unlike antibiotics, their specificity limits side-effects such as damage to the physiological microbiota [10]. Phages are ubiquitous in environments where bacteria proliferate, coevolving with bacteria and contributing to the regulation of their population, thereby maintaining equilibrium in ecosystems [11]. However, one of the main concerns associated with phage applications is the potential swift emergence of phage-bacterial variants that exhibit resistance or reduced susceptibility to phages, which may jeopardize the success of such applications, as occurs with antibiotics [12,13]. Within this framework, the PhagoVet consortium, established in 2018 through funding from H2020-FTI call is dedicated to registering a bacteriophage-based product (PhagoVet) for *Salmonella* control in poultry farming. In this study, we investigated the emergence of *S*. Infantis variants resistant to or with reduced susceptibility to PhagoVet in broilers because of the increasing impact of *S*. Infantis in poultry production. To our knowledge, the emergence of these variants had not been previously studied in this bacterium.

## 2. Materials and Methods

### 2.1. Bacterial Strains 

*Salmonella enterica* serovar Typhimurium LB5000 (SGSC181; University of Calgary, Calgary, Canada) and *Salmonella enterica* serovar Enteritidis LK5 (SGSC3820; University of Calgary, Calgary, Canada) strains were used to propagate and quantify the bacteriophages. A chromosomal spontaneous mutant resistant to rifampicin (Rif^R^) was obtained from the *S.* Infantis 1724105 strain and was employed for challenging *Gallus gallus* in farm trials. The *S*. Infantis 1724105 strain was obtained from a broiler farm as part of Salmonella self-controls following Regulation (EC) 2160/2003 (from Centro de Calidad Avícola y Alimentación Animal de la Comunidad Valenciana, CECAV, Castellón, Spain). All *Salmonella* strains were cultured in Luria–Bertani (LB) broth, agar plates, or XLD agar (Xylose-Lysine-Deoxycholate Agar; Becton Dickinson, Heidelberg, Germany) media, supplemented with rifampicin (75 μg/mL) when required. In all cases, plates were incubated for 18 h at 37 °C.

### 2.2. PhagoVet Product

PhagoVet is a bacteriophage-based product developed by a European consortium integrated by ALS (Tondela, Portugal), Vetworks (Poeke, Belgium), CECAV (Alquerias, Castellón, Spain) and UAB (Barcelona, Spain). It consists of four virulent bacteriophages (UAB_1, UAB_60, UAB_69, and UAB_Phi78), selected from our *Salmonella* phage library, with production for this study conducted by Jafral (Ljubljana, Slovenia). The PhagoVet product was prepared by mixing the lysates of the four bacteriophages to obtain a titer of 1 × 10^10^ PFUs/mL. Phage titration was performed by plating ten-fold serial dilutions onto LB plates using the double agar method and the appropriate bacterial host [14].

### 2.3. Host Range Determination of the PhagoVet Product

The lysis ability of the cocktail was tested against 271 *Salmonella* strains of the serovars Agona, Anatum, Derby, Enteritidis, Hadar, Heidelberg, Infantis, Kentucky, Mbandaka, Mikawasima, monophasic Typhimurium, Newport, Ohio, Saintpaul, Senftenberg, Stanley, Typhimurium, and Virchow. The methodology used for this study was the spot test onto bacterial lawns, as reported [14].

### 2.4. Bacteriophage Characterization and Genome Sequencing

Transmission electron microscopy (TEM) was employed to determine the bacteriophage morphologies as previously described [15]. For genome sequencing, high-titer lysates (10^11^–10^12^ PFUs/mL) were obtained by ultracentrifugation at 51,000× *g* for 2 h, and DNA was purified using the phenol-chloroform method [16]. Sequencing and preliminary analysis of the sequences was performed by STAB VIDA (Caparica, Portugal) on the Illumina MiSeq platform, using 300 bp paired-end sequencing reads and an average sequencing depth of 100×. The analysis of the generated sequence raw data was carried out using CLC Genomics Workbench 12.0. (Qiagen, Redwood City, CA, USA). The trimmed sequence reads were used to perform a de novo assembly approach using an algorithm based on de Bruijn graphs [17] and a preliminary annotation was performed using the pipeline from RAST server version 2.0 (Rapid Annotation using Subsystem Technology) (http://rast.nmpdr.org/; accessed on 25 June 2019) [18]. Different analyses of the phage genomes were performed using Geneious 2020.0.5. (Biomatters, Auckland, New Zealand.). Firstly, BLAST was performed, and the closest hits were searched. From this analysis, the phage genus that they belonged to and the model phage of the specific genus were searched on the ICTV web page (https://talk.ictvonline.org/; accessed on 2 February 2020). ProgressiveMAUVE [19] was used for genome comparisons at the nucleotide level with their respective model phages, and the genomes were zeroed using those phages as references. When required, a manual search to identify open-reading frames (ORF) was conducted using BlastX. Functional predictions were conducted using BLASTp programs [20], HMMscan (https://www.ebi.ac.uk/Tools/hmmer/search/hmmscan, accessed on 5 May 2020) and eggNOG [21]. 

Furthermore, in silico analyses of bacteriophage genomes were carried out using the Virulence Factor Database (VFDB, http://www.mgc.ac.cn/VFs/; accessed on 21 July 2021) [22] to identify virulence-associated genes, and ResFinder [23] and the CARD database [24] were employed to detect antibiotic resistance genes. The VIRIDIC program facilitated the taxonomic classification [25].

### 2.5. Isolation of S. Infantis Variants with Reduced Phage Susceptibility 

The *S*. Infantis 1724105 Rif^R^ strain used in animal trials is sensitive to two of the PhagoVet phages (UAB_60 and UAB_69). Therefore, this study focused on determining the emergence of bacterial variants resistant or with reduced susceptibility to these two phages in two animal trials using broilers. In both trials, the minimum number of animals per group ensuring independent replicates and enough data for conducting appropriate statistical analysis were used. Furthermore, the *Salmonella*-free status on the arrival of the animals was corroborated in cloacal samples from 25% of the animals. After each trial, poultry farms were emptied, washed, and disinfected for the next trial round. All trials adhered to Regulations (EC) 1831/2002 and 429/2008, according to the additive use, animal categories involved, and following advice on the adequate statistical power. All experimental procedures involving the handling of experimental animals were approved by the Ethical Review Panel of the Directorate-General for Agriculture, Fisheries and Livestock from the Valencian Community, by the code 2021/VSC/PEA/0003, according to Spanish regulations (Real Decreto 53/2013) [26]. In the following paragraphs, the experimental procedure of each trial is detailed, and the design is summarized in Table S1. 

Trial 1. A total of 288 male one-day broilers were purchased from a local commercial source and located in two independent rooms separated by walls within the same barn to avoid cross-contamination with phages and *Salmonella*. Two different groups were assessed: T1 (positive control challenged with *Salmonella*) and T2 (challenged with *Salmonella* and treated with a minimum PhagoVet dose). Each group had 12 replicates with 12 animals per replicate (n = 144 animals/group). On arrival and after randomization to treatments, broilers of both groups received water and were fed ad libitum from day 1 to the end of the trial. After 24 h of rearing, 20% of the birds in both groups were orally challenged with *S*. Infantis 1724105 Rif^R^ at a concentration on 10^4^ CFUs/animal. PhagoVet product was administered through individual drinkers via drinking water once a week at a dose of 10^6^ PFUs/animal. 

The isolation of *Salmonella* variants with reduced susceptibility to UAB_60 and/or UAB_69 phages involved the collection of feces with boot swabs from T1 and T2 groups on days 3, 21, and 42 of bacterial infection. From each experimental group, a pool of feces was prepared. For this, each sample was diluted 1:10 in buffered peptone water (BPW) followed by homogenization. Subsequently, 1 mL of each individual sample was added to a flask and thoroughly mixed. To isolate *Salmonella* colonies from each pool, 10-fold serial dilutions in 0.9% NaCl buffer were prepared and plated on XLD agar plates supplemented with rifampicin (75 µg/mL). After overnight incubation at 37 °C, the *Salmonella* concentration was calculated. Afterward, a maximum of 200 colonies for each time and group were randomly selected and isolated on LB plates supplemented with rifampicin (75 µg/mL). To ensure the absence of contaminating bacteriophages, each isolate was streaked on green plates three times [27]. Finally, the colonies were streaked on LB plates supplemented with rifampicin (75 µg/mL) and incubated at 37 °C for 20 h. The susceptibility of *Salmonella* isolates to UAB_60 and UAB_69 bacteriophages was determined as previously described [28]. In all assays, the *S*. Infantis Rif^R^ parental strain was included as a control. In those cases where a minimal number of colonies grew on the counting plates, all were individually isolated, and their sensitivity to phages was subsequently determined.

Trial 2. The design of this trial closely mirrored Trial 1, with the presented following changes. Thus, following 24 h of rearing, 50% of birds within groups T1 and T2 (n = 144 animals/group) were orally challenged with *S*. Infantis 1724105 Rif^R^ at a concentration of 10^6^ CFUs/animal. The PhagoVet product was administered at a dosage of 10^8^ PFUs/animal via the drinking water on three occasions during the first week (upon the broiler’s arrival, 24 h post-infection with *S*. Infantis, and 24 h after the second PhagoVet administration). Thereafter, the product was administered weekly through individual drinkers during the second and third weeks. In addition, the animals of the T2 group underwent a 2 h period of water fasting upon arrival to the farm (i.e., before the first product application) to guarantee optimal PhagoVet consumption at the proper dose. To identify the *Salmonella* variants, the procedure was like in Trial 1 with the following modifications. Two distinct pools, one comprising 12 boot swabs (feces) and the other consisting of 10 ceca, were made on days 7, 14, and 21 of infection from the T1 and T2 groups. The *Salmonella* concentration was determined following the procedure outlined in Trial 1, and a maximum of 200 colonies per time point and type of samples were isolated to search for the desired variants. 

For both trials, *Salmonella* enumeration from cecum samples (24 cecum samples from each experimental group in both T1 and T2 trial) was performed at the end of the trials by the miniaturized most probable number technique previously described (ISO/TS 6579-2:2012) [29]. Furthermore, zootechnical parameters, such as body weight (BW), mortality and feed rate conversion (FRC), were assessed. 

### 2.6. Statistical Analysis 

Each trial described above is a completely randomized design, with pen as the experimental unit for statistical purposes. Results of the ISO/TS 6579-2:2012 [29] were treated by one-way ANOVA using the General Linear Model (GLM) function in SPSS Statistics Software (IBM, v.27, IBM Corp: Armonk, NY, USA). Differences due to phage treatment in the performance parameters during the study were evaluated using a GLM. All parameters have been reported as group least squares mean. Standard error of the mean, difference of the mean and 95% confidence intervals have also been reported. Significant differences have been declared at *p* ≤ 0.05.

## 3. Results

### 3.1. Characteristics of PhagoVet Product

**Figure 1:**
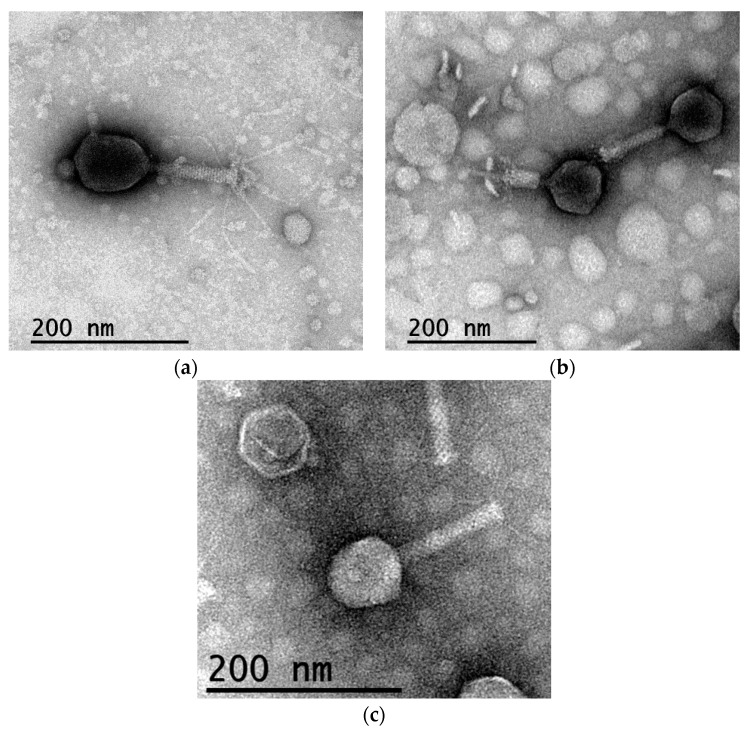
Electron micrographs of bacteriophages UAB_1 (**a**), UAB_60 (**b**), and UAB_69 (**c**). Scale bars are detailed in the images.

The PhagoVet product is composed of the UAB_1, UAB_60, UAB_69, and UAB_Phi78 bacteriophages. As the UAB_Phi78 bacteriophage had been previously characterized [15,30], we proceeded to study the other three phages at both microscopic and genomic levels. As depicted in Figure 1, the UAB_1 bacteriophage features an icosahedral head (92.7 ± 2.7 nm) and a contractile tail (108.2 ± 2.1 nm). Similarly, UAB_60 exhibited an identical morphology, with a head measuring 112.0 ± 6.3 nm and a tail of 115.9 ± 2.7 nm, while UAB_69 possessed a head of 74.7 ± 2.1 nm and a tail of 113.9 ± 4.2 nm. The genomes of UAB_1, UAB_60, and UAB_69 bacteriophages were sequenced, and their complete genomes were deposited in the Genbank database under accession numbers OL656106, OL656107, and OL656108, respectively. Genomic analysis of UAB_1, UAB_60, and UAB_69 revealed their affiliation with the *Justusliebigvirus*, *Tequatrovirus*, and *Felixounavirus* genera, respectively. UAB_Phi78 belonged to the *Zindervirus* genus, as previously reported [30]. The genomes of UAB_1 and UAB_69 bacteriophages exhibited short direct terminal repeats (DTR) (Figure S1), while the genome of UAB_60 lacked DTR. Furthermore, the in silico analyses of the genomes revealed no similarities to known virulence-associated genes or antibiotic resistance genes. In addition, no genes encoding potential immunoreactive food allergens or genes suggesting factors associated with lysogeny were identified. 

On other hand, it is noteworthy that the PhagoVet product demonstrated a broad host range against 271 strains encompassing 18 *Salmonella* serovars, as shown in Table 1.

### 3.2. Identification of S. Infantis Variants with Reduced PhagoVet-Susceptibility 

In both trials, the animals remained generally healthy throughout the study, with no observed abnormal clinical signs. 

Data from Trial 1 showed that the overall mortality rate was 4.86%, with no significant differences between treatment groups T1 and T2 (*p* > 0.05). Similarly, there were no significant differences observed in body weight (T1: 2418.9 vs. T2: 2354.4; *p* > 0.05) or feed conversion ratio (FCR) (T1: 2.00 vs. T2: 1.53; *p* > 0.05) at 42 d of the study. In this trial, *S*. Infantis was administered at a low infective dose (10^4^ CFUs/animal), resulting in a *Salmonella* concentration in the feces of the challenge group of approximately 5.7 log_10_ CFUs/g, maintained at 6.1 log_10_ CFUs/g until at least 21 d and reaching 4.6 log_10_ CFUs/g by the end of the study (42 d) (Table 2). Furthermore, treatment with PhagoVet (10^6^ PFUs/mL) led to a reduction in the *Salmonella* concentration by approximately 1 log_10_ CFUs/g at 21 d, reaching nearly undetectable levels by the end of the study (T2 group, 42 d). However, the *Salmonella* concentration in the ceca, as determined by the MPN, was <1 log_10_ CFUs/g at the end of the study in both groups (Table S2). To assess the presence of variants with reduced susceptibility to UAB_60 and UAB_69 bacteriophages, a total of 600 and 413 colonies were isolated from the feces of T1 and T2 groups, respectively (Table 2). After susceptibility testing, all of them were found to be sensitive to both phages.

In Trial 2, the overall mortality rate was 3.1%, with no significant differences between treatment groups T1 and T2 (*p* > 0.05). Likewise, there were no significant differences observed in body weight (T1: 2598.72 vs. T2: 2611.43; *p* > 0.05) or feed conversion ratio (T1: 2.19 vs. T2: 2.00; *p* > 0.05) at 42 d of the study. In this trial, the *Salmonella* infective dose and the PhagoVet dose were 10^6^ CFUs/animal and 10^8^ PFUs/animal, respectively, and the PhagoVet administration schedule was modified. Samples for *Salmonella* counting in both feces and ceca were taken at 7, 14 and 21 d. A total of 600 colonies from the feces of T1 and T2 groups, respectively, and 457 and 564 colonies from the ceca of T1 and T2 groups, respectively, were isolated for studying phage susceptibility. Results revealed that the bacterial concentration in feces was in general lower than those observed in trial 1 for both T1 and T2 groups (Table 3). However, higher *Salmonella* counts were detected in ceca at day 7 for both groups (Table 3), decreasing to very low values at 21 d (Table 3), regardless of phage treatment. The reduction in the cecal *Salmonella* population was corroborated by the most probable number technique at the end of the study (day 42). As observed in Trial 1, the concentration of *Salmonella* in the ceca was below 1 log_10_ CFUs/g in both T1 and T2 groups (Table S2). In this trial, variants with reduced susceptibility to phages were identified (Table 4). Specifically, in the feces, one of the 200 clones isolated at 7 d from the T1 group exhibited reduced susceptibility to UAB_69 phage. The same was observed for clones isolated at 14 d and 21 d from the T2 group. However, all these variants remained sensitive to the UAB_60 phage. Among cecum isolates, only five at 7 d from the T1 group demonstrated reduced susceptibility to both UAB_60 and UAB_69 phages.

## 4. Discussion

There is significant concern about the potential emergence of resistant variants or those with reduced susceptibility to phages, which may compromise their application in phage therapy and other uses. Aware of this problem, along with the increasing incidence of *S*. Infantis infections in both humans and animals and understanding the advantages of applying phages in avian production for controlling *S*. Infantis, we studied the impact of the emergence of this type of bacterial variants in oral phage therapy in broiler production administering the PhagoVet product, a cocktail composed of four bacteriophages.

Microscopic characterization and genomic analysis of the UAB_1, UAB_60, UAB_69 and UAB_Phi78 bacteriophages showed that all of them belonged to the *Caudoviricetes* class, but to different genera within this class (Figure 1) [30]. The genomes of UAB_1 and UAB_69 bacteriophages exhibited short direct terminal repeats (DTR) (Figure S1), like the UAB_Phi78 genome [30]. In contrast, bacteriophage UAB_60 did not have DTR. This phage belonged to the *Tequatrovirus* genus, which includes T4-like bacteriophages, whose packaging mechanism results in terminase cleavage at random sites, leading to genome termini with permutations [31]. This explains why we did not find terminal ends of the genome. On the other hand, the wide host range of the PhagoVet product (Table 1) and the absence of negative genomic determinants in the phage genomes support that the PhagoVet product can be considered safe and suitable for application in animal production. In this respect, the PhagoVet product efficiently reduced *S*. Infantis in feces around 1 log_10_ at 21 d (Trial 1) and achieved almost undetectable values at the end of the experiment (reduction > 4 log_10_) (Table 2). 

The experimental conditions of infection of the broilers with *S*. Infantis allowed this bacterium to persist in feces until the end of Trial 1 (Table 2), reaching concentrations in the intestinal tract comparable to those reported by other authors during similar experimental periods [32,33]. However, its concentration remained below 1 log_10_ CFUs/g in ceca using the NMP method at the end of both trials (Table S2). This could be attributed to the fact that, in our case, only a small percentage of the animals was orally challenged with *Salmonella* or also to the characteristics of the *Salmonella* strain used in these works. We ruled out the absence of the pESI-like mega-plasmid or the virulence genes encoded within it as a contributing factor, as genome sequencing confirmed their presence in the *S*. Infantis 1724105 strain. Nevertheless, it must be noted that other authors encountered similar problems of *Salmonella* colonization of the gastrointestinal tract, even administering *Salmonella* by oral gavage [34]. Furthermore, it has been reported that changes in the gastrointestinal microbiota, which can reduce available resources or produce certain metabolic products, could adversely impact the growth and survival of *Salmonella* strains used in challenge experiments, particularly over a two-week period of experimentation [35,36,37]. Despite the difficulties in demonstrating phage therapy efficacy in cecal content, our results regarding *Salmonella* reduction in feces are comparable to those obtained in other studies, albeit with other *Salmonella* serovars, performed in similar experimental periods [32,34], and, to our knowledge, this study is the first to use oral phage therapy in broilers infected with *S*. Infantis.

Regarding the emergence of variants with reduced susceptibility to phages, it must be highlighted that none of these variants were detected in Trial 1. In Trial 2, in which the *Salmonella* infective dose was highest, one of them was detected in feces at 7 d from the untreated group, and one at 14 and 21 d from the treated group (Table 4). From the ceca, only five variants were isolated at 7 d from the untreated group. We speculate that these five variants could be clonal, and that variants found could either be present in the bacterial cultures used for animal infection or have arisen spontaneously during the early days of the infection when the *Salmonella* concentration in the intestine was highest. In any case, these variants failed to colonize the digestive tract of the broilers because the *Salmonella* concentration in the ceca was extremely low at 42 d (Table S2). It must be noted that the total number of variants was similar in both untreated and treated groups, suggesting that there was no effect of the phage treatment on the emergence of these variants. This finding aligns with a previous study conducted by us with broilers and *S*. Typhimurium [28]. 

Previous works have reported dissimilar results concerning the detection of phage resistance. A review on the development of bacteriophage resistance during bacteriophage therapy revealed that phage-resistant variants of different bacterial species emerged in up to 80% of studies targeting the intestinal tract (out of 11 studies) on different animal species and in 50% of studies (out of 6) using sepsis models on mice [38]. Interestingly, the intestinal tract seemed more susceptible to the emergence of phage-resistance, and although in some circumstances it has been associated with the alteration of known virulence factors, such as O-antigen or LPS [38], it seems more likely to be linked to target bacteria acquiring genes encoding mechanisms to interfere with the phage multiplicative cycle through horizontal transfer from the abundant intestinal microbiota [28]. Despite the increasing number of studies on the use of bacteriophages in animal production, few have been carried out on *Gallus gallus* and *Salmonella*, specifically exploring the emergence of bacterial variants with reduced susceptibility [33,34,36,39]. One study demonstrated that phages reduced the cecal colonization of *S. enterica* serovars Enteritidis and Typhimurium in broilers, at least within 4 days of treatment [39]. The authors isolated bacteriophage-insensitive mutants able to colonize chicken ceca within 24 to 48 h of phage treatment, but these mutants were not maintained for extended periods in ceca. Hurley et al. [36] performed a trial for 30 days on *S*. Typhimurium-infected broilers without a clear reduction in *Salmonella* levels in feces. They found phage-resistant mutants at 15 and 29 days in animals irrespective of phage administration. More recently, two studies conducted with *S*. Typhimurium and *S*. Enteritidis did not find resistant variants in feces and cloacal swabs at 35 days [34] and in caeca at 42 days of trials [33]. In fact, it seems that bacterial resistance to phages often entails a fitness cost [38,40], although this may not consistently result in reduced infectivity, at least in the intestinal tract [38].

## 5. Conclusions

This is a pioneering study applying phage therapy against *S.* Infantis under conditions that closely mirror those encountered in broiler production. The absence of phage-resistant variants or those with reduced susceptibility following the administration of the PhagoVet product highlights its potential effectiveness in reducing or eliminating *S.* Infantis on poultry farms.

The product led to a significant reduction in *S.* Infantis concentrations in feces, demonstrating its potential as a control measure. Furthermore, even in broilers with low levels of intestinal colonization by *S.* Infantis, the PhagoVet product did not give rise to the emergence of resistant bacterial variants, suggesting that the risk of compromising the efficacy of this treatment is minimal.

## Figures and Tables

**Table 1:** Percentage of infection of the PhagoVet product of *Salmonella* strains of different serovars.

Serotypes	Number of Strains per Serotype	PhagoVet (%) ^a^
Monophasic Typhimurium	20	100
Agona	22	100
Anatum	12	100
Derby	10	100
Enteritidis	24	100
Hadar	19	100
Heidelberg	4	100
Infantis	53	96
Kentucky	15	93
Mbandaka	12	100
Mikawasima	7	86
Newport	5	100
Ohio	12	92
Saintpaul	1	100
Senftemberg	11	36.4
Stanley	2	100
Typhimurium	24	92
Virchow	18	72
Total	271	93

^a^ The values are the percentage (%) of the strains of each serotype infected by the PhagoVet product.

**Table 2:** *S.* Infantis Rif^R^ concentration and number of colonies isolated from feces over time in Trial 1.

Time (Day)	T1		T2	
	Concentration (log_10_ CFUs/g)	No. Isolates Tested	Concentration (log_10_ CFUs/g)	No. Isolates Tested
3	5.7	200	5.6	200
21	6.1	200	5.2	200
42	4.6	200	nc	13 ^a^

T1, group of animals challenged with *Salmonella*. T2, group of animals challenged with *Salmonella* and treated with PhagoVet. nc, not calculated, the number of colonies per plate was lower than 15. ^a^, all colonies that grew on count plates were tested.

**Table 3:** *S.* Infantis Rif^R^ concentration in both feces and broiler ceca over time in Trial 2.

Time (Day)	*Salmonella* Concentration (log_10_ CFUs/g)			
	Feces		Ceca	
	T1	T2	T1	T2
7	4.5	3.7	6.7	6.7
14	4.9	5.3	3.7	3.1
21	4.4	4.8	nc	nc

T1, group of animals challenged with *Salmonella*. T2, group of animals challenged with *Salmonella* and treated with PhagoVet. nc, not calculated because the number of colonies per plate was lower than 15.

**Table 4:** *S.* Infantis RifR variants isolated in Trial 2 from feces and broiler ceca with reduced susceptibility to UAB_60 and UAB_69 bacteriophages.

Time (Day)	T1				T2			
	No. Tested Isolates	No. Variants (%)			No. Tested Isolates	No. Variants (%)		
		UAB_60	UAB_69	UAB_60 and UAB_69		UAB_60	UAB_69	UAB_60 and UAB_69
Feces								
7	200	0	1 (0.5)	0	200	0	0	0
14	200	0	0	0	200	0	1 (0.5)	0
21	200	0	0	0	200	0	1 (0.5)	0
Ceca								0
7	200	0	0	5 (2.5)	200	0	0	0
14	200	0	0	0	200	0	0	0
21	57 ^a^	0	0	0	164 ^a^	0	0	0

T1 group, animals infected with *Salmonella*. T2 group, animals infected with *Salmonella* and treated with PhagoVet. ^a^, all colonies that grew on count plates were tested.

## Data Availability

Data are available upon request.

## Supplementary Materials

The following supporting information can be downloaded at https://www.mdpi.com/article/10.3390/ani14162352/s1: Figure S1: Sequences of the short direct terminal repeats identified in the genomes of UAB_1 and UAB_69 bacteriophages; Table S1: Details of the animal trials; Table S2: *S*. Infantis Rif^R^ counts in broilers ceca (log_10_ CFUs/g) by the end of study in Trials 1 and 2, determined by the NMP method.
