# Supplementary material for: Limited Emergence of Salmonella enterica Serovar Infantis Variants with Reduced Phage Susceptibility in PhagoVet-Treated Broilers
Source: Animals (Basel). 2024 Aug 14;14(16):2352. doi: 10.3390/ani14162352 (PMC11350853; doi:10.3390/ani14162352)
Supplement: Supplementary file 1 [file animals-14-02352-s001.zip › animals-3137948-supplementary.pdf]

A) Direct terminal repeat of UAB_1 bacteriophage
5′-
AGCCTACTTTCTTAAAAGCGCAATAAATCAGAATTATCTTGATTTTTAGGGCGTTTTATAGTACAGAA
AGTATGCAAATGATAATCATTATCAGCTATCTTCCACGGGTGGCACTATTTCACGCCAGATCATTTCA
TTTGGCAAGGTGTATATAGATGTTTAGAAATGCCAAGCCATTGATTTACTTAGAATTTATCTGTTTTG
TTGTGAGTAATGCCTTTCTTGCTCAGAAAATAATCAAAAGTCTTATAAGTCATTGAATGATAATGCA
AATGATAATTATTCTCATTGATATTTGAGGGGGACGGTCGGCAGAAATCGGGGGGCGCTGAAAATC
GCGTTA-3′

B) Direct terminal repeat of UAB_69 bacteriophage
5′-
TAGCCCCCTTAACATTTTCTTAACAATTTCTTAACAATCCCTACATAGTTATGCTTTTAAGGCACTTGT
TAGCAACTTGTTAGCCCCTTGTTAAATCTCTGTTAAGACAACGTAAAAGCCTTGTAAGAGTCTTTGAC
ATAGCCTTAAAAGCTCCTGTAAAGCTCTATAAAAAGGTGTCTGTACAGTTGGGGTGTAACTACTAGG
GAGAGGCTTAAAAGGGCTTACAAAGAGCTATAGAAAGCTTTTAAAAGGTATGTTTAAGGAATCACT
AAGAAAGGAAAGGGAACGATAAGAAAAGGGATAGTGACCATGTTAATAGCTTGTTAAAAAGATTA
GACACTTGTTAGGAACTTGTTAATTATCTTAACAAAATAACTCTTAAAAATTAACTAATTGGTATTGT
TACCAATCCCTGTTAGCTCTTGTTAGTCATCTGTTAAGAGACTATAAAGACCATGTATCGACTGTTAA
GAACTTGTTACAGCCTTGTTAGTTCTGTTAAGAATCTGTTAAGACATCTGAAGAGATGTTAAGAGCTT
GAGAGAGTTGTTAAGAGATTGTTAATGATATTTTAAAATTCTGCAAAGGGGATGTTACAGGGATGTT
AAGGGCTACCAA-3′

Figure S1. Sequences of the short direct terminal repeats identified in the genomes of UAB_1 and UAB_69 bacteriophages.

Table S1. Details of the animal trials.

Trial number	Group	ID	PhagoVet	*Salmonella*
1	Untreated Group	T1	-	10^4 CFU/bird
	Treated group	T2	10^6 PFU/bird/week	10^4 CFU/bird
	Nº Treatments: 2<br>Nº pen replicates/treatment: 12		Nº broilers/treatment: 144<br>Total nº of pens: 24	Total nº animals: 288<br>Nº animals/pen: 12
	Animals: broiler males<br>Duration of the trial: 42 days		Age of the animals: 1 day<br>End point: week 6	
2	Untreated Group	T1	-	10^6 CFU/bird
	Treated group	T2	10^8 PFU/bird/week*	10^6 CFU/bird
	Nº Treatments: 2<br>Nº pen replicates/treatment: 12		Nº broilers/treatment: 144<br>Total nº of pens: 24	Total nº animals: 288<br>Nº animals/pen: 12
	Animals: broiler males<br>Duration of the trial: 42 days		Age of the animals: 1 day<br>End point: week 6	

*Except for the first week of life, where the administration of the PhagoVet product occurred upon the broilers' arrival, 24 h post-infection, and 24 h after the second PhagoVet product administration.

Table S2. *S.* Infantis RifR counts in broilers ceca (log_{10} CFU/g) by the end of the study in Trials 1 and 2, determined by the NMP method.

Trial	Group	Mean	SE	95% IC		*P-value*
				Lower	Upper	
1	T1	0.32	0.27	-0.21	0.84	0.33
	T2	0.68	0.27	0.16	1.21	
2	T1	0.22	0.035	0.16	0.29	>0.05
	T2	0.29	0.038	0.22	0.36	

T1, group of animals infected with *Salmonella*

T2, group of animals infected with *Salmonella* and treated with PhagoVet.

SE, standard error.
